# Secondary Hyperalgesia Phenotypes Exhibit Differences in Brain Activation during Noxious Stimulation

**DOI:** 10.1371/journal.pone.0114840

**Published:** 2015-01-23

**Authors:** Mohammad Sohail Asghar, Manuel Pedro Pereira, Mads Utke Werner, Johan Mårtensson, Henrik B. W. Larsson, Jørgen Berg Dahl

**Affiliations:** 1 Department of Anaesthesia, Centre of Head and Orthopaedics, Rigshospitalet, Copenhagen University Hospitals, Copenhagen, Denmark; 2 Multidisciplinary Pain Centre, Neuroscience Centre, Rigshospitalet, Copenhagen University Hospitals, Copenhagen, Denmark; 3 Max Planck Institute for Human Development, Berlin, Germany; 4 Department of Psychology, Lund University, Lund, Sweden; 5 Functional Imaging Unit, Hospital, Glostrup, Copenhagen University Hospitals, Glostrup, Denmark; University of South California, UNITED STATES

## Abstract

Noxious stimulation of the skin with either chemical, electrical or heat stimuli leads to the development of primary hyperalgesia at the site of injury, and to secondary hyperalgesia in normal skin surrounding the injury. Secondary hyperalgesia is inducible in most individuals and is attributed to central neuronal sensitization. Some individuals develop large areas of secondary hyperalgesia (high-sensitization responders), while others develop small areas (low-sensitization responders). The magnitude of each area is reproducible within individuals, and can be regarded as a phenotypic characteristic. To study differences in the propensity to develop central sensitization we examined differences in brain activity and anatomy according to individual phenotypical expression of secondary hyperalgesia by magnetic resonance imaging. Forty healthy volunteers received a first-degree burn-injury (47°C, 7 min, 9 cm^2^) on the non-dominant lower-leg. Areas of secondary hyperalgesia were assessed 100 min after the injury. We measured neuronal activation by recording blood-oxygen-level-dependent-signals (BOLD-signals) during mechanical noxious stimulation before burn injury and in both primary and secondary hyperalgesia areas after burn-injury. In addition, T1-weighted images were used to measure differences in gray-matter density in cortical and subcortical regions of the brain. We found significant differences in neuronal activity between high- and low-sensitization responders at baseline (before application of the burn-injury) (*p* < 0.05). After the burn-injury, we found significant differences between responders during noxious stimulation of both primary (*p* < 0.01) and secondary hyperalgesia (*p* ≤ 0.04) skin areas. A decreased volume of the right (*p* = 0.001) and left caudate nucleus (*p* = 0.01) was detected in high-sensitization responders in comparison to low-sensitization responders. These findings suggest that brain-structure and neuronal activation to noxious stimulation differs according to secondary hyperalgesia phenotype. This indicates differences in central sensitization according to phenotype, which may have predictive value on the susceptibility to development of high-intensity acute and persistent pain.

## Introduction

Injury-induced sensitization of the central nervous system is a condition that is believed to play an important role in the development and maintenance of pain. During sensitization the central nervous system is regulated into a state of high reactivity in which sensory inputs from both injured and non-injured adjacent areas produce augmented responses [1–3]. One important question is whether some individuals develop more central sensitization than others and consequently, if such individuals are predisposed to higher levels of acute and chronic pain experience.

A number of experimental models are used in the study of central sensitization in volunteers [1,2]. Noxious stimulation of the skin with either electrical [3], chemical [4] or heat [5,6] stimuli leads to the development of primary hyperalgesia at the site of injury, and to secondary hyperalgesia in normal skin surrounding the injury [1,2]. The proneness to develop secondary hyperalgesia has been used to study differences in central sensitization [7]. Some individuals develop large secondary hyperalgesia areas (high-sensitization responders) while others only develop small secondary hyperalgesia areas (low-sensitization responders) [7]. Although the magnitude of secondary hyperalgesia areas varies widely between individuals, the intra-individual variability is low, i.e., individuals will continue to develop areas of similar magnitude when exposed to the same noxious stimulus [7]. Development of secondary hyperalgesia thus seems to be a phenotypic characteristic [7].

No studies have yet compared high- and low-sensitization responders regarding differences related to central pain processing. Development of secondary hyperalgesia areas of different magnitudes may reflect variations in brain anatomy and brain function, i.e., cerebral pain processing. Understanding these variations is important to gain further insight into central mechanisms of pain and to investigate, if such variations are predictive of the severity of acute pain and the propensity for transition to chronic pain.

Magnetic resonance imaging (MRI) gives a unique opportunity to assess microstructural differences in brain anatomy [8] while blood-oxygenation-level-dependent (BOLD) functional magnetic resonance imaging (fMRI) is widely used to map the neuronal dependent hemodynamical responses to pain in humans [9,10].

The aim of the present exploratory study was therefore to compare functional and structural characteristics of brain activity and anatomy in high- and low-sensitization phenotypes using (f)MRI. We hypothesized that according to phenotype differences in the activity and the anatomy of the brain could be demonstrated.

## Methods

### Volunteers

We recruited volunteers through advertisements in a magazine for university students and through own records from completed studies. Inclusion and exclusion criteria are presented in (Table 1). Based on a previous study [11] the interquartile range of secondary hyperalgesia areas was 24–55 cm^2^. We therefore defined high-sensitization responders as volunteers developing secondary hyperalgesia areas above 55 cm^2^ and low-sensitization responders as volunteers developing secondary hyperalgesia areas below 24 cm^2^.

**Table 1:** Inclusion and exclusion Criteria.

**Inclusion Criteria**	**Exclusion Criteria**
ASA I-II	The volunteer is not cooperative
20 yrs ≤ age ≤ 35 yrs	The volunteer does not understand or speak Danish or English
18 kg/m^2^ < BMI < 30 kg/m^2^	Pregnancy, breastfeeding, women planning pregnancy or not using contraceptives (pill or IUD)
Urine sampled negative for amphetamines, barbiturates, benzodiazepines, cocaine, opioids (buprenorphine, methadone, morphine) and tetrahydrocannabinol (THC)	Coffee, tea, cocoa or other methylxanthine-containing foods or beverages and tobacco for at least 12 h before start of the study
Negative pregnancy test	No development of secondary hyperalgesia area 100 minutes after a first-degree burn
Secondary hyperalgesia area < 24 cm^2^ or Secondary hyperalgesia area > 55 cm^2 §^	Participation in a drug trial in the previous 60 days
	Alcohol or drug abuse
	Use of psycho-active drugs or analgesics
	Use of prescription drugs 1 week before the trial
	Use of over-the-counter medication 48 hours before the test
	Neurological condition
	Chronic pain condiion
	Allergy to morphine or naloxone
	Skin lesions in the assessment areas
	Signs of neuropathy in the ipsilateral or contralateral assessment areas
	Contra-indication to MRI scanner
	Volunteer refuses to be informed in case of pathological findings in the MRI scans.

§ based on a previous study [11] the interquartile range of secondary hyperalgesia areas was 24–55 cm^2^. We therefore defined high-sensitization responders as volunteers developing secondary hyperalgesia areas above 55 cm^2^ and low-sensitization responders as volunteers developing secondary hyperalgesia areas below 24 cm^2^.

All volunteers gave written consent after receiving detailed oral and written information. The volunteers were paid EUR 200 (USD 260) as a compensation for their participation. The study took place on two study days, a screening day, and an experimental day separated by at least one week. The study was approved by the Committee on Health Research-Ethics of Copenhagen (H-3-2012-117) and the Danish Data Protection Agency (30–0871 / 01994) and was performed according to the principles of the Helsinki Declaration with later revisions.

### Screening day

Screening and assessments took place in a quiet room at the Multidisciplinary Pain Center, Rigshospitalet, from Monday to Sunday between 08.00 AM and 08.00 PM. The first inclusion date was 17.10.12. The screening included information about the trial, gathering of a brief medical history, and a physical examination. Volunteers completed the Hospital Anxiety and Depression Scale (HADS)[12] and the Pain Catastrophizing Scale (PCS) [13]. Volunteers then received a first-degree burn-injury. Pain during the burn, as well as areas of secondary hyperalgesia were assessed.

#### Induction of secondary hyperalgesia

The first degree burn-injury was induced with a contact thermode (Thermotest, Somedic AB, Hörby, Sweden [2.5 × 5.0 cm^2^, 47.0°C,7 min]), with the upper anterior corner 11 cm below the medial meniscus margin and 6 cm from the anterior margin of the tibia in the dominant leg. Pain during the burn injury was assessed with a numeric rating scale (NRS, 0 = no pain; 10 = worst imaginable pain)[14]. The secondary hyperalgesia area was assessed after 100 min[15] using a polyamide monofilament (Aesthesiometer nominal value 18 (bending force (mean ± SD): 937 ± 14 mN); Somedic AB) by stimulating in 8 diagonal and orthogonal lines converging towards the center of the burn-injury. Volunteers were instructed to close their eyes during the assessments. The stimulations started in skin with normal sensation. When the volunteers reported the occurrence of a definite change in sensation (uncomfortable feeling, burning or stinging sensation), this was defined as the border of the secondary hyperalgesia area. The corners of the octagon were marked on the skin and transferred to a transparent sheet. The size of the hyperalgesia area (cm^2^) was calculated using a computer-based vector-algorithm (Canvas 12.0, ACD Systems International, Victoria, Canada).

### Experimental Day

The experimental procedures, including MRI scans took place at the Radiology Department, Glostrup Hospital, from Monday to Sunday between 08.00 AM and 08.00 PM. The first MRI scan session was conducted 04.11.12 while the last MRI scan session was completed on the 11.03.13. A blood sample was collected to for measurements of hematocrit, hemoglobin and electrolytes. The burn injury location and procedure, including assessment of secondary hyperalgesia areas, were similar to the screening day, except that the non-dominant leg, and a 9 cm^2^, MRI compatible TSA-II contact thermode (3.0 × 3.0 cm^2^, Medoc Ltd, Ramat Yishai, Israel) was used for testing (two volunteers, (one high- and one low-sensitization responders respectively) were tested on the right side (non-dominant side) while the remaining volunteers were tested on the left side).

### Assessment of mechanical pain thresholds

**Figure 1:**
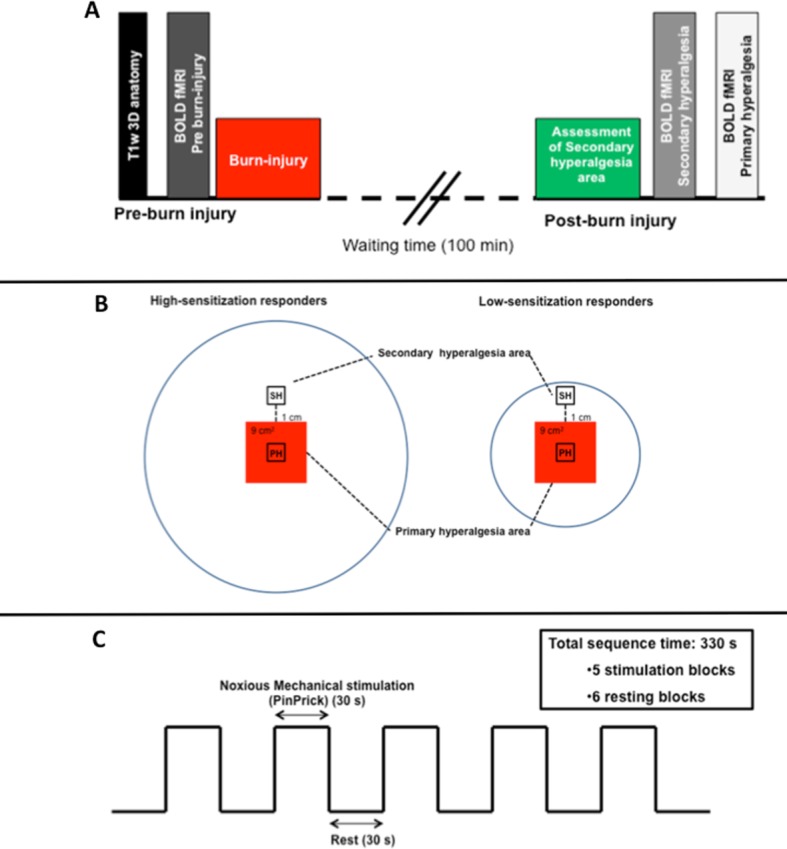
A: Study Algorithm – Experimental day (Top). All variables were recorded in fixed order throughout the study. MRI was performed on a 3.0 T Philips Achieve Scanner (Philips Medical Systems, Best, The Netherlands) using a 32-channel phased-array send and receive head coil. Initially T1-weighted 3D anatomical images were obtained. Pre-burn BOLD response measurements were performed during painful pin-prick stimulation. Subsequently a first-degree burn-injury was induced on the non-dominant lower leg. After 100 min waiting period the area of secondary hyperalgesia was assessed. The volunteers were then re-positioned in the scanner and BOLD response measurements were repeated twice. There was at least 5 min pause between each BOLD response measurement. **B: Stimulation areas (Middle)**. Areas of noxious pin-prick mechanical stimulation in high- and low-sensitization responders. High-sensitization responders (left) developed large areas of secondary hyperalgesia surrounding the burn-injury site (red square) while low-sensitization responders only developed small areas of secondary hyperalgesia (right). In the *first* BOLD response measurement, pin-prick stimulation was performed inside the secondary hyperalgesia area one cm from the border of the burn-injury (SH). In the *second* BOLD response measurement, pin-prick stimulation was performed inside the primary hyperalgesia area (PH). Pin-prick stimulations were delivered within a predetermined area of 1×1 cm. Pain was rated on a verbal numerical rating scale (NRS) after each stimulation. **C: Stimulation paradigm (Buttom)**. To obtain BOLD fMRI measurements we applied noxious mechanical stimulation by weighted-pin stimulators (pin-prick) in a block design. Pain thresholds were individually assessed by pin-prick stimulation and the lightest pin-prick stimulator perceived as painful before induction of the burn-injury was used throughout the study. The stimulation paradigm consisted of rest blocks with no stimulation, alternating with active blocks of pin-prick (pain) stimulation delivered at 1 Hz. One scan session thus consisted of abbrev6 resting blocks (30 s/block) interleaved by 5 stimulation blocks (30 s/block) with a total duration of 5 min 30 sec.

Mechanical pain thresholds were assessed using weighted-pin stimulators (PinPrick, MRC Systems, Heidelberg, Germany (8, 16, 32, 64, 128, 256, 512 mN)) with a contact area of 0.049 mm^2^ according to Dixon’s “up-and- down” method [16]. Assessment area was the non-dominant lower leg, where the burn injury was later induced. Before burn injury the volunteers were stimulated 5 times and were asked to indicate when more than 2 of the pin-prick stimulations were perceived as painful. Using pin-prick stimulators of ascending or descending order, the thresholds were determined 5 times and the median of these assessments was then considered for analysis. We used the lightest pin-prick stimulator, which was perceived as painful, for all subsequent pin-prick stimulation sequences during BOLD recordings throughout the study. For experimental procedure and timings (Fig. 1).

### Experimental procedure

MRI was performed on a 3.0 T Philips Achieve Scanner (Philips Medical Systems, Best, the Netherlands) using a 32-channel phased-array send and receive head coil. Initially a reference anatomical whole-brain image was obtained, followed by T1-weighted 3D anatomical images.

Volunteers were positioned in the MRI scanner in a comfortable supine position. Supportive soft cushions were used to limit head movement. Monitoring included continuous ECG, end-tidal CO_2_, and respiratory rate, while non-invasive arterial blood pressure was measured intermittently at pre-determined fixed intervals.

Pre-burn injury BOLD response measurements were performed during painful pin-prick stimulation. Following induction of the burn injury, and a waiting period of 100 min secondary hyperalgesia areas were assessed. The subjects were then re-positioned in the scanner and BOLD response measurements were repeated twice (Fig. 1). In the first BOLD response measurement, painful pin-prick stimulation was performed inside the secondary hyperalgesia area one cm from the border of the burn injury. In the second BOLD response measurement, pin-prick stimulations were performed inside the primary hyperalgesia area (Fig 1B). Pin-prick stimulations were delivered within a predetermined area of 1 x 1 cm. Pain was rated on a numerical rating scale (NRS: 0–10) after each stimulation. There was at least 5 min pause between each BOLD-response measurement. Pain was rated on a NRS after each stimulation.

## Data Acquisition and Imaging Protocols

### Anatomical Images

Anatomical images were acquired using a T1-weighted 3D turbo field echo sequence (137 sagittal slices 1.1 mm thick; in-plane resolution 1.1 × 1.1 × 1.1 mm: repetition time 6.9 ms; echo time 2.8 ms; flip angle 9°).

### BOLD response

BOLD functional imaging utilized a gradient echo EPI sequence (33 slices 4.0 mm thick; slice gap 0.1 mm; field of view 230 × 230 × 134 mm; in-plane acquired resolution 1.8 × 1.8 × 4.0 mm; repetition time 3.0 s; echo time 35 ms; flip angle 90°; SENSE factor 2). Slices were oriented parallel to the inferior border of corpus callosum covering the whole brain. The 110 dynamic scans were obtained during each 5.5 min scan session.

To record BOLD response, we applied mechanical stimulation with pin-pricks. The paradigm consisted of six rest blocks (30 s/block), in which no stimulation was performed with alternating five active blocks (30 s/block), where pin-prick stimulation was performed at 1 Hz with a total scan time of 5 min 30 sec per BOLD response measurement (Fig. 1). Volunteers were told to stay awake and asked to keep their eyes open during the BOLD scans.

## MRI Data Analysis

### BOLD fMRI preprocessing and statistical analysis

Functional images were analyzed using FMRIB software Library (FSL) version 5.0.1, Oxford, UK (http://www.fmribox.ac.uk/fsl). FMRI Expert analysis Tool (FEAT) was used for pre-processing (first level analysis). The first two volumes of each functional scan were removed to allow equilibration of image intensities. Pre-processing steps included motion-correction, brain extraction, spatial (5 mm) smoothing, and temporal filtering (high pass 100 s). The block stimulation paradigm convolved with a two-gamma hemodynamic response function served as a model time course. Whole brain analysis was performed on the data using general linear model (GLM). Statistical results were co-registered first to the subject’s own T1-weighted 3D anatomical images and subsequently to the standard Montreal Neurological Institute atlas (MNI-152) using linear registration, full search and 12 degrees of freedom (except in #21 were co-registration was done directly to MNI-152).

The 3D anatomical images had prior to this been transformed to match the dimension of the functional scans using FSLreorient2sted. Subjects that were left-handed (N = 2; one high- and one low-sensitization responder) were right-left swapped using FSLswapdim. Brain extraction was performed using FSL brain extraction tool (BET) (Fractional intensity threshold: 0.6, threshold gradient of 0.1, and robust brain center estimation).

A full quality assurance (QA) was completed, which included individual visual inspection to ensure that all subjects had a full set of scans including pre-burn injury, secondary hyperalgesia scan and primary hyperalgesia scan. Furthermore it was ensured that there was no excess motion (>3 mm) and an optimal co-registration. All scans passed the QA were included in the subsequent analysis.

Individual GLM results were then fed into a fixed-effects analysis model (second level analysis) using FLAME (FMRIB’s Local Analysis of Mixed Effects) and outlier de-weighting. In addition analysis was repeated with gender added as a covariate to rule out possible gender related confounding of the results.

Z (Gaussianised T/F) statistical images were thresholded using clusters determined by Z > 2.3 and a corrected cluster significance threshold of *p* = 0.05, corrected for multiple comparison by false discovery rate [17]. We analyzed differences between high- and low-sensitization responders at each stimulation zone separately using an unpaired t-test. BOLD changes are presented as Z scores (Δ (x – mean)/standard deviation)) and p values for high sensitization responders compared to low sensitization responders. Z scores of ≥ 1.96 corresponding to a p value of ≤ 0.05 were determined as significant activation in a region. Individual regions were identified using Juelich Histological Atlas [18] and in addition verified by translating to corresponding anatomical structures using GingerALE 2.3.1 and Talaraich Daemon 2.4.2.

### Structural MRI preprocessing and statistical analysis

Anatomical images were preprocessed using the FreeSurfer imaging analysis suite (http://surfer.nmr.mgh.harvard.edu/; version 5.3). FreeSurfer is a semi-automatic software package that performs volumetric segmentation of subcortical structures [19–21].

Subcortical volume estimates were exported to IBM SPSS Statistics (version 21) and a Pearson correlation analysis was performed to assess the relationship between regional subcortical volume and secondary hyperalgesia areas. All volumes where adjusted for total intracranial volume based on the analysis of covariance approach as outlined in Raz et al. [22]. This was performed to avoid possible confounding due to differential head-size between participants.

### Statistics

Our primary endpoint was differences in BOLD responses before and after induction of the burn injury between high- and low-sensitization responders. Secondary endpoints were differences in brain structure between high- and low-sensitization responders. Further, data on HADS- and PCS-assessments, pain during burn-injuries, areas of secondary hyperalgesia, and mechanical pain thresholds, were analyzed.

Preliminary estimation of sample size was based on detection of differences in BOLD response between the two groups. We wanted to detect a 3% difference between high and low-sensitization responders with regard to BOLD response to noxious mechanical stimulation (minimal relevant difference). Under the assumption that data is normally distributed and using a significance level (α) of 0.01, β = 0.2, inter-individual standard deviation in BOLD response (σ) = 2.5%, the estimated necessary number of subjects is 32. However, in order to compensate for any drop-outs, we set the number of volunteers to 40 (i.e. 20 in each group).

Data were assessed for normality using Q-Q plots and Shapiro-Wilk W test. In case of normally distributed data, we used unpaired t-test to compare mechanical pain thresholds, pain scores (NRS) and psychometric scales between high- and low-sensitization responders, whereas Mann-Whitney rank sum test was used for non-normal data. Secondary hyperalgesia areas in the screening and the experimental days were compared with Wilcoxon rank sum test.

Data are given as mean (SD) or median (25–75% interquartile range (IQR)).

## Results

A total of 55 volunteers were screened for magnitude of secondary hyperalgesia areas. Two volunteers did not develop a measurable secondary hyperalgesia area, while nine did not meet the area requirements to be classified as high- or low-sensitization responders. Two volunteers suffered from claustrophobia and one had to be excluded due to concurrent illness. In total 14 volunteers were excluded after the screening day prior to inclusion. Seven volunteers (# 26, 28, 30, 31, 35, 37, 38) were considered low-sensitization responders, although they developed a slightly larger secondary hyperalgesia area in the screening Day than the cut-off value of 24 cm^2^. However, in the experimental day, these seven volunteers had smaller secondary hyperalgesia areas than 24 cm^2^ and were therefore included in the study.

In total, 41 volunteers were included in the study. On the experimental day, one volunteer (BNK) was not able to stay awake during the scan sessions and was therefore excluded. Hence, per-protocol data from 40 healthy volunteers (22 F, 18 M) were included (#1–20: high-sensitization responders; #21–40: low-sensitization responders) A T1-weighted anatomical scan from one subject had to be removed due to misalignment during acquisition (#21). Two subjects (#11 and #35) did not complete the HADS and PCS questionnaires.

Demographic data are shown in (Table 2). Detailed individual demographic data are shown in (S1 Table). There were no significant differences between high-sensitization and low-sensitization responders with regard to age, sex, height or weight (*p* > 0.05); however, there was a trend towards a larger number of females than males (Fisher’s exact test *p* = 0.11) in the high-sensitization group.

**Table 2:** Demographic Data.

	**High-sensitization responders**	**Low-sensitization responders**
**Male/Female (n)**	6 / 14	12 / 8
**Age (years)**	24.3 ± 2.3	24.2 ± 2.6
**Height (cm)**	175.3 ± 9.3	177.9 ± 11.5
**Weight (kg)**	68.7 ± 8.5	71.5 ± 10.3

Mean values ± SD.

Blood pressure, heart rate and end-tidal CO_2_ parameters recorded during MRI scans are shown in (Table 3). Detailed individual hemodynamic data are shown in (S2 Table)Baseline blood chemistry showed normal hematocrit, potassium- and sodium levels except in the few cases with marginally borderline values. There were no differences between high- and low-sensitization responders with regard to any of the blood chemistry parameters (*p* > 0.05). For blood samples outside the normal range (Table 4).

**Table 3:** Blood pressure (BP), heart rate and end-tidal CO_2_ during fMRI BOLD scans (Mean + SD).

	**BP systolic (mm Hg)**	**BP diastolic (mm Hg)**	**Heart rate (/ min)**	**End tidal CO_2_ (mm Hg)**
**High-sensitization responders**				
**Pre-Burn scan**	120.8 ± 8.8	68.0 ± 6.9	62.7 ± 7.9	4.7 ± 0.4
**Secondary hyperalgesia scan**	123.7 ±10.6	66.7 ± 5.7	63.5 ± 8.1	4.6 ± 0.6
**Primary hyperalgesia scan**	124.9 ± 10.4	69.7 ± 6.3	65.4 ± 8.8	4.7 ± 0.5
**Low-sensitization responders**				
**Pre-burn scan**	118.8 ± 8.3	66.3 ± 10.4	58.9 ± 12.0	5.0 ± 0.3
**Secondary hyperalgesia scan**	123.4 ± 12.3	62.3 ± 7.2	60.0 ± 10.7	5.0 ± 0.3
**Primary hyperalgesia scan**	122.0 ± 9.3	66.9 ± 7.5	64.3 ± 10.9	4.9 ± 0.3

**Table 4:** Baseline blood samples.

**Blood sample**	**Normal values**	**Volunteer**	**Blood chemistry**
Haematocrit	[M: 0.41–0.50]	#8 M:	0.40
	[F: 0.35–0.46]	#20 F:	0.30
Haemoglobin	[M: 8.4–10.8 mmol/]	#8 M:	8.2
	[7.2–10.0 mmol/L]	#5 F:	7.1
		#20 F:	6.8
Potassium	[3.5–5.0 mmol/L]	#24 F:	3.3
		#26 M:	3.4
		#39 F:	3.4
Sodium	[135–145 mmol/L]	#14 F:	147
		#32 M:	148
		#34 F:	146

Baseline blood chemistry showed normal hematocrit, potassium- and sodium levels except in the few cases shown in this table with marginally borderline values. There were no differences between high- and low-sensitization responders with regard to any of the blood chemistry parameters (*p* > 0.05).

### BOLD responses

#### Pre-burn assessments

**Figure 2:**
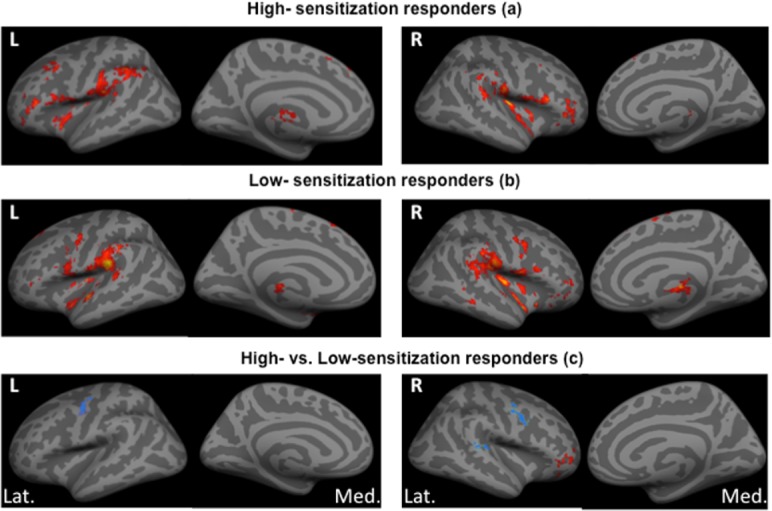
Brain activation during mechanical noxious stimulation before burn-injury. Group analysis showing brain activation by BOLD fMRI during mechanical noxious stimulation at baseline (pre-burn) in (a) high-sensitization responders and (b) low-sensitization responders. (c) Shows differences in brain activation between high- and low-sensitization responders. Activity is displayed upon standardized inflated brain figures with red areas showing regions-of-interest (ROI) with increased or positive activation and blue areas showing ROI’s with decreased or negative activation. We found significant differences in brain activation with more activation in the right inferior frontal gyrus and less activation in the left superior temporal gyrus, left, and right precentral gyrus in high- versus low-sensitization responders. L = Left side, R = Right side, Lat. = Lateral view, Med. = Medial view.

(Fig. 2A and 2B) shows the neuronal activation maps while (Table 5 and Table 6) shows the z-stats scores and coordinates for neuronal activation following noxious mechanical stimulation before burn-injury in high- and low-sensitization responders, respectively.

**Table 5:** Pre-burn group activation to noxious mechanical stimulation in high-sensitization responders.

**Brain Region**	**Structure**	**Lat.**	**z-stat**	**X (mm)**	**Y (mm)**	**Z (mm)**	**Vol (cm3)**
**Positive Activation**							
***Cortical***							
*Frontal*							
Middle	*BA 6, 8, 9*	L	5.99	−48	12	42	21.24
Medial	*BA 6, 8*	L	4.46	0	32	40	06.75
Inferior	*BA 10, 46, 47*	R	4.09	48	50	−4	06.77
*Parietal*							
Post-central	*BA 3,1,2, 40, 41,42*	L	5.99	−52	−24	16	33.94
Secondary Somato-sensory	*BA 5,7*	L	5,99	−36	−24	18	05.45
*Temporal*							
Superior	*BA 21, 22, 38*	R	5.99	56	2	−2	18.60
*Insular*							
Insula	*BA 13, 41*	R	5.99	36	−20	14	26.31
***Subcortical***							
Cingulate	*BA 30,31,32*	L	5.99	−5	38	22	12.51
Thalamus	*VL*	L	2.75	−6	−18	0	04.13
Thalamus	*VL, VPL, MD, VA*	R	2.24	14	−12	6	03.36
***Brainstem / Cerebellum***							
Culmen		L	1.75	−2	−50	−2	02.76
Pyramis		R	5.99	30	−70	−30	24.73

**BA:** Broadmann area, **VL:** Ventral Lateral Nucleus, VPL: Ventral Posteriolateral Nucleus, **MD:** Medial Dorsal Nucleus, **VA:** Ventral Anterior Nucleus.

**Table 6:** Pre-burn group activation to noxious mechanical stimulation in low-sensitization responders.

**Brain Region**	**Structure**	**Lat.**	**z-stat**	**X (mm)**	**Y (mm)**	**Z (mm)**	**Vol (cm3)**
**Positive activation**							
***Cortical***							
*Frontal*							
Superior	*BA 6*	L	2.45	−6	26	62	03.65
Medial	*BA 6*	L	1.97	−8	−6	68	03.02
Inferior	*BA 10, 46*	R	4.59	46	52	−4	05.09
*Parietal*							
Postcentral	*BA 3,1,2,4 6*	L	1.93	−52	−8	50	02.97
Secondary somato sensory	*BA 5,7*	L	5,99	−42	−15	18	03.23
*Temporal*							
Superior	*BA 40,41,42*	L	2,45	−46	−24	11	56.7
*Insula*							
Insula	*BA 13*	R	5.99	62	−32	20	76.31
*Subcortical*							
Cingulate	*BA 31,32*	L	2.45	−4	35	18	07.14
Thalamus	*MD, VPM, VL, VPL*	R	5.99	12	−18	4	08.24
***Brainstem / Cerebellum***							
Cerebellar Tonsil		R	5.17	28	−64	−34	11.07
Cerebellar Tonsil		L	5.04	−28	−62	−36	10.42

**BA:** Broadmann area; **MD:** Medial dorsal nucleus; **VPM:** Ventral Posteriomedial nucleus, **VL:** Ventral Lateral nucleus, **VPL:** Ventral posteriolateral nucleus.

We recorded less neuronal activation in the ipsilateral precentral gyrus (left, *p* = 0.046) and ipsilateral superior temporal gyrus (left, *p* = 0.04) after noxious stimulation of the normal skin (before burn-injury) in high- versus low-sensitization responders. In addition, after correction for gender, we recorded increased activation of the contralateral inferior frontal gyrus (right, *p* = 0.0049) and less activation of contralateral precentral gyrus (right, *p* = 0.00047) in high- versus low-sensitization responders (Fig. 2C), (Table 7).

**Table 7:** Contrast analysis results before burn injury in high- versus low-sensitization responders.

**Brain Region**	**Structure**	**Lat.**	**z-stat**	**X (mm)**	**Y (mm)**	**Z (mm)**	**Vol (cm^3^)**
**Positive activation**							
***Cortical***							
*Frontal*							
Inferior	*BA 10, 46,47*	R	2.58	44	52	−2	03.73
**Negative activation**							
***Cortical***							
*Frontal*							
Precentral	*BA 4,6,46*	R	3.31	26	−18	62	05.03
Precentral	*BA 4,6*	***L***	***2.61***	−50	−4	42	***03.32***
			2.91				07.44
*Temporal*							
Superior	*BA 40,42, 43*	L	1.97	−68	−22	14	02.75

Contrast analysis results for noxious mechanical stimulation in high-sensitization responders versus low-sensitization responders for noxious mechanical stimulation before burn injury. Bold italic letters shows results without gender correction, while the results after correction for gender are written in normal letters. **BA:** Broadmann area

#### Post-burn assessments

(Table 8 and 9) show the z-stats scores and coordinates for neuronal activation after induction of the burn injury in the secondary and primary hyperalgesia areas.

**Table 8:** Contrast analysis results for noxious stimulation inside the secondary hyperalgesia in high- vs. low-sensitization responders.

**Brain Region**	**Structure**	**Lat.**	**z-stat**	**X (mm)**	**Y (mm)**	**Z (mm)**	**Vol (cm^3^)**
**Positive activation**							
***Cortex***							
*Parietal*							
Post-central	*BA 2, 40*	R	3.62	42	−30	34	05.79
Precuneus	*BA 5,7*	R	***1.74***	14	−48	66	***02.73***
			1.83				02.79
Precuneus	*BA 7, 4, 5*	L	1.99	2	−44	66	02.97
***Subcortical***							
Posterior Cingulate	*BA 23,29,30*	L	4.32	2	−54	12	07.70
Caudate		R	5.99	−36	−14	40	35.52
Parahippocampal	*BA 30*	R	3.01	30	−58	6	04.48

Contrast analysis results for noxious mechanical stimulation inside the secondary hyperalgesia areas for high-sensitization responders versus low-sensitization responders. Letters in bold italic show results without gender correction, while the results after correction for gender are written in plain. **BA:** Broadmann area

**Table 9:** Contrast analysis results for noxious stimulation inside the primary hyperalgesia areas in high- vs. low-sensitization responders.

**Brain Region**	**Structure**	**Lat.**	**z-stat**	**X (mm)**	**Y (mm)**	**Z (mm)**	**Vol (cm^3^)**
**Negative Activation**							
***Cortex***							
*Frontal*							
Precentral	BA 4,6	L	***3.30***	−48	−8	58	***04.60***
			3.30				04.77
***Brainstem / cerebellum***							
Cerebellum anterior lobe		L	2.91	−8	−50	−26	04.02

Contrast analysis results for noxious mechanical stimulation inside the primary hyperalgesia areas for high-sensitization responders versus low-sensitization responders. Letters in bold italic show results without gender correction, while the results after correction for gender are in plain. **BA:** Broadmann Area.

**Figure 3:**
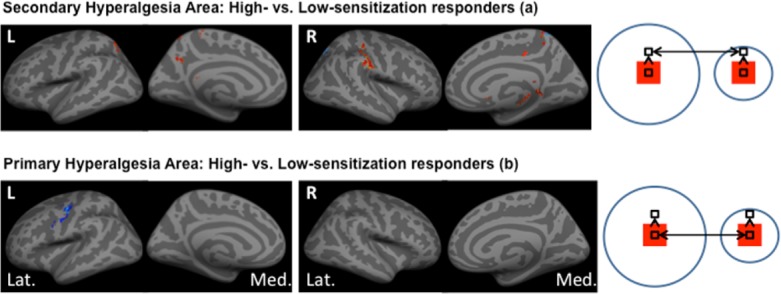
Differences in brain activation between high- and low-sensitization responders during post-burn mechanical noxious stimulation. Group analysis showing differences in brain activation by BOLD fMRI between high- and low-sensitization responders. Activity is displayed upon standardized inflated brain figures with red areas showing regions-of-interest (ROI) with increased activation and blue areas showing ROI’s with decreased activation. The cartoon on the right illustrates the stimulation zones for the pin-prick stimulation. **A: Secondary hyperalgesia**: We found significant differences in brain activation between high- and low-sensitization responders after stimulation inside the secondary hyperalgesia area with more activation of the right post-central gyrus, left precuneus, left posterior cingulate cortex, right parahippocampal gyrus, right caudate nucleus and less activation of right precuneus in high- compared to low-sensitization responders. **B: Primary hyperalgesia**: After stimulation inside the primary hyperalgesia areas we found less activation in the left precentral gyrus and anterior lobe of cerebellum *(not shown)*.

We recorded less activation of the contralateral precuneus (right, *p* = 0.041) after noxious stimulation in the secondary hyperalgesia area in high- versus low-sensitization responders. After correction for gender we in addition recorded increased activation in the contralateral post-central gyrus (right, *p* = 0.00015), ipsilateral precuneus (left, *p* = 0.023), ipsilateral posterior cingulate cortex (left, *p* < 0.0001), contralateral parahippocampus gyrus (right, *p* = 0.0013) and contralateral caudate nucleus (right, *p* < 0.0001) in high- versus low-sensitization responders (Fig. 3A).

We recorded less activation in the ipsilateral precentral gyrus (left, *p* = 0.00048) after noxious stimulation in the primary hyperalgesia area in high- versus low-sensitization responders. Correcting for gender lead to less recorded activation in the ipsilateral anterior lobe of the cerebellum (left, *p* = 0.0018) (Fig. 3B).

### Structural MRI results

**Figure 4:**
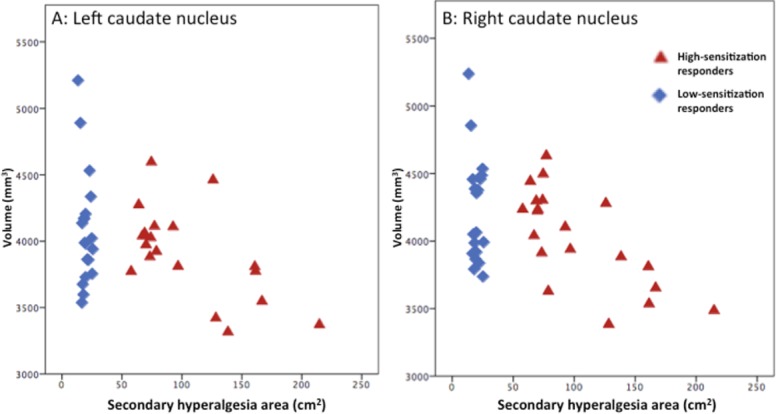
Structural differences between high- and low-sensitization responders. Volumes of caudate nuclei are shown as a function of secondary hyperalgesia areas in high-sensitization responders (red triangles) and low-sensitization responders (blue diamonds). High-sensitization responders showed smaller volume of the left (*p* = 0.001) and right (*p* = 0.01) (corrected p-values) nucleus caudate volume compared to low-sensitization responders.

We recorded a smaller volume of the caudate nuclei in subjects with large secondary hyperalgesia areas, with a negative correlation between adjusted caudate volume and secondary hyperalgesia area. This was true for both hemispheres and mean secondary hyperalgesia areas: Right caudate nucleus; r = −0.498, *p* = 0.001; Left caudate nucleus; r = −0.407, *p* = 0.01; all n = 39, (Fig. 4). There were no differences between men and women regarding the volume of the caudate nuclei (*p* > 0.05). There where no effects in other brain regions (all, *p >* 0.05)

### Pain during burn injury

During induction of the burn-injury both groups reported moderate to severe pain (NRS = 6.0 ± 1.8 in high-sensitization responders vs. NRS = 5.8 ± 2.1 in low-sensitization responders (*p* = 0.72)). Erythema and hyperalgesia were observed in all volunteers following the burn-injury. For detailed individual results (S3 Table).

### Areas of secondary hyperalgesia

**Figure 5:**
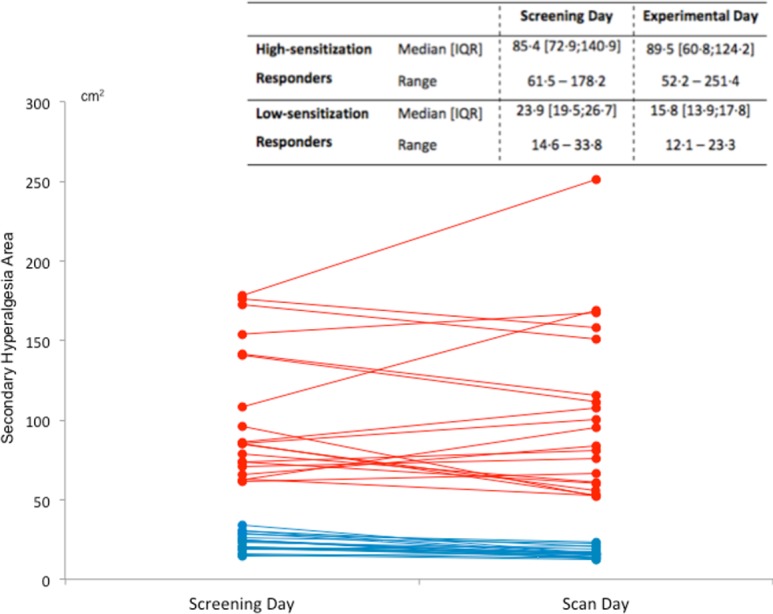
Secondary hyperalgesia areas: Secondary hyperalgesia areas assessed 100 min after a first-degree burn-injury in the screening day and in the experimental day in high- and low-sensitization responders. The table shows the median, inter quartile range (IQR) and range of secondary hyperalgesia areas in high- and low-sensitization responders on the screening day and experimental day respectively. Red lines show the magnitude of secondary hyperalgesia areas in high-sensitization responders, while blue lines show the magnitude of secondary hyperalgesia areas in low-sensitization responders. There were no differences in secondary hyperalgesia areas between the two experimental days in high-sensitization responders (*p* = 0.71). In low-sensitization responders the secondary hyperalgesia areas were smaller on the experimental day by a median (experimental day – screening day) of −5.4 cm^2^ (interquartile range; −9.8 to −3.6 cm^2^) (*p* < 0.01). This difference could however be subscribed to differences in thermode size between the screening and the experimental days. On the screening day a 12.5 cm^2^ thermode was used while a smaller MRI compatible thermode of 9 cm^2^ was used on the experimental day.

High-sensitization responders developed secondary hyperalgesia areas with a mean of 102.9 + 41.2 cm^2^ (range: 61.5–178.2 cm^2^) on the screening day, and of 103.4 ± 52.6 cm^2^ (range: 52.2–251.4 cm^2^) on the experimental day (*p* = 0.71). Low-sensitization responders developed secondary hyperalgesia areas with a mean of 23.1 ± 5.5 (range: 14.6–33.8 cm^2^) on the screening day, and of 16.5 + 3.2 cm^2^ (range: 12.1–23.3 cm^2^) on the experimental day. The areas were smaller on the experimental day by a mean of 6.7 ± 5.3 cm^2^ (*p* < 0.01) in these responders (Fig. 5). A smaller, (MRI compatible) thermode was, however, used on the experimental day (9 cm^2^), compared to the screening day (12.5 cm^2^).

### Mechanical pain thresholds

**Figure 6:**
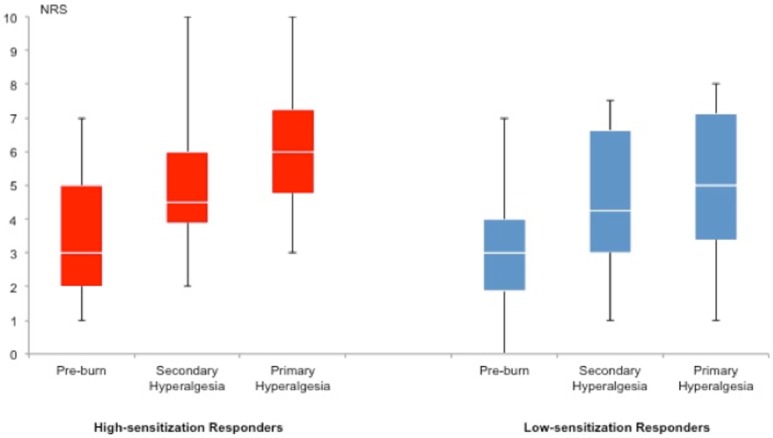
Pain ratings after noxious mechanical stimulation. Boxplot of pain ratings by numeric rating scale (NRS) in high- (red boxes) and low-sensitization responders (blue boxes). Noxious mechanical stimulation by pin-prick did not result in any differences in pain scores between high- and low-sensitization responders before burn-injury (high-sensitization responders: 3.6 ± 1.9; low-sensitization responders 2.9 ± 1.8; *p* = 0.29). After burn-injury there were no differences in pain scores after stimulation in the secondary hyperalgesia areas (high-sensitization responders: 5.0 ± 2.1, low-sensitization responders: 4.4 ± 2.1 (*p* = 0.37)) or after stimulation in the primary hyperalgesia areas (high-sensitization responders: 6.3 ± 2.1, low-sensitization responders 5.1 ± 2.2, *p* = 0.08).

There was no difference in mechanical pain thresholds between the groups *(p* = 0.27). The median pin-prick force used was 128 mN (interquartile range [IQR] 128–256) in high-sensitization responders and 256 mN (IQR 128–512) in low-sensitization responders. There was no difference in pain scores between high- and low-sensitization responders either before the burn-injury (*p* = 0.29, normal skin) or after the burn injury following stimulation in the secondary hyperalgesia area (*p* = 0.37), or primary hyperalgesia area (*p* = 0.08). (Fig. 6) illustrates NRS values from both high- and low-sensitization responders during pin-prick stimulation before and after the burn-injury. For detailed individual results (S4 Table)

### HADS and PCS

In the HADS questionnaire we observed a trend towards higher scores in the anxiety subscale in the high- compared to the low-sensitization responders (*p* = 0.08). When comparing females in the high-sensitization group with females in the low-sensitization group in regard to anxiety scores, significantly higher scores were observed in the high-sensitization group (*p* = 0.007; Mann-Whitney-test). A similar comparison for males did not demonstrate any significant difference (*p* = 0.32; Mann-Whitney-test). In agreement, comparing gender differences among the high-sensitization responders in regard to anxiety scores, higher values for females than males, were observed (*p* = 0.009; Mann-Whitney-test). No gender difference was observed in a similar comparison among the low-sensitization responders (*p* = 0.29; Mann-Whitney-test). One female (#13) had an anxiety score of 15 indicating a clinical significant anxiety trait.

Although a trend of higher rumination scores (PCS; *p* = 0.03) was observed in high- vs. low-sensitization responders, no differences were seen in other PCS-variables or the global PCS-score (*p* = 0.17). No gender-related differences in PCS-scores were observed. For detailed individual results (S5 Table).

## Discussion

Injury-induced sensitization of the central nervous system is believed to play a crucial role in the development and maintenance of pain [23,24]. One essential feature of central sensitization is secondary hyperalgesia, i.e. expansion of receptive fields or recruitment of wide dynamic range neurons in the spinal cord enabling input from non-injured tissue to be perceived as painful [23,24]. Secondary hyperalgesia is expressed differently among individuals, and has been suggested to be a phenotypic characteristic [7].

The major findings of this study were that to a uniform noxious mechanical stimulation healthy volunteers showed dissimilarities in neuronal activation associated with their propensity to develop large or small areas of heat-induced, secondary hyperalgesia without reporting significantly increased pain scores. The observed differences increased after gender-wise correction. An inverse relationship between the magnitude of secondary hyperalgesia, and the volume of the caudate nuclei was further demonstrated. Finally, we recorded a trend towards higher anxiety scores in high- versus low-sensitization responders, reaching significance in females.

### Pre-burn cerebral differences between phenotypes

We found that high-sensitization responders had a smaller volume of the right and left caudate nuclei in comparison with low-sensitization responders. Further, we recorded more activation of the contralateral inferior frontal gyrus and less neuronal activation bilaterally in the precentral gyrus and in the ipsilateral superior temporal gyrus, after noxious stimulation of normal skin in high- versus low-sensitization responders.

An inverse relationship between pain sensitivity and gray matter density in cortical regions has been demonstrated in earlier studies [25]. A lower volume of the caudate nucleus has been recorded in pain conditions such as fibromyalgia [26] and chronic fatigue [27], but is also a known feature in various psychiatric diseases including depression [28,29]. It has been suggested that the caudate nuclei are sites for integration of sensory, motor, and motivational processes into behavioral output [30]. The caudate nuclei play an important role in sensory processing [31] and suppression of pain [32]. Specifically activation of the caudate nuclei is an important element in spatial discrimination of pain [31] and in addition contributes to suppression of motor responses to pain stimuli [33].

One possible interpretation of our results is that high-sensitization responders have a comparatively attenuated sensory discrimination [34], are less effective at suppressing pain and furthermore, are more prone to avoidance behavior due to diminished suppression of nociceptive motor responses. This is confirmed by the pre-burn functional results. The decreased activation of the motor cortices may suggest that high-sensitization responders have more prepotent pain avoidance behavior. Consequently, stronger suppression is necessary to avoid excessive motion during the MRI measurements. The inferior frontal gyrus has been implicated in inhibition of response behavior (Go/No-Go tasks) [35], indicating an active suppression of motor responses resulting in the globally decreased motor cortex activity. The superior temporal gyrus deals with emotional aspects of pain [36] and is responsible for the integration of previous experiences in decision-making [37].

### Post-burn cerebral differences between phenotypes

After stimulation in the secondary hyperalgesia area we recorded more activation in the caudate nuclei, parahippocampus gyrus, post-central gyrus on the contralateral side and in the precuneus and posterior cingulate cortex on the ipsilateral side; while less activation was recorded in the contralateral precuneus in high- versus low-sensitization responders. After stimulation in the primary hyperalgesia area we recorded less activation in the ipsilateral precentral gyrus and anterior lobe of the cerebellum in high- versus low-sensitization responders.

The differences in neuronal activation evoked by stimulation of the hyperalgesic skin further confirm that high- and low-sensitization responders process pain differently. The “default mode network” is a defined system of brain areas that is active when individuals are not focused on external environment [38]. Nociceptive stimulation of secondary hyperalgesia areas induces strong deactivations in brain areas belonging to the default mode network [39]. We recorded differences in brain areas related to the default mode network (precuneus, posterior cingulate cortex), with increased (less reduced) activations in high-sensitization responders and more attenuated activation in low-sensitization responders. Interestingly, chronic pain patients also demonstrate less deactivation in this network [40].

The post-central gyrus (primary somato-sensory cortex) is a part of the sensory-discriminative dimension of pain [41]. Increased activation has been observed in allodynia [42] and has been correlated with increased pain sensitivity [43]. Interestingly activation has been observed in allodynia [42] and unilateral increased activation has been associated with chronic pain [44]. The parahippocampal gyrus is associated with pain memory [45]. Activation of cerebellum in pain studies is generally considered to be primarily associated with pain avoidance reflecting a latent motor response [46]. Decreased activation thus suggests a suppression of withdrawal-movements, due to the experimental settings not allowing the volunteers to move within the MRI scanner.

Our results have confirmed previous observations that the area of secondary hyperalgesia is a robust reproducible phenomenon [7], thus asserting that healthy subjects can be phenotyped with regard to secondary hyperalgesia areas. We have furthermore demonstrated differences between phenotypes with regard to brain anatomy and central pain processing at rest and after induction of secondary hyperalgesia without reporting concomitant differences in pain scores. These findings suggest differences in pain sensitivity between groups. Previous studies have suggested a correlation between secondary hyperalgesia area and persistent pain [47,48] although one study failed to find an association between areas of secondary hyperalgesia and acute postoperative pain [49]. The area of secondary hyperalgesia is believed to be dependent on individual predispositions [7,47] rather than correlated to pain thresholds at rest or injury-related factors [47]. Specifically we have reported similarities between high-sensitization responders and chronic pain patients with regard to brain activation. One hypothetical interpretation could therefore be that these subjects have a higher propensity to develop central sensitization and therefore are at higher risk for developing chronic pain.

### Impact of gender

There was a trend towards a larger number of females than males in the high-sensitization responders group. Intracranial volume varies between sexes due to differences in body size [50]. While gender-specific regional differences in brain anatomy have been demonstrated, there are no differences with regard to the caudate nuclei volume between sexes when adjusted for intracranial volume [51,52]. This is confirmed in the present study. In addition, in the analysis of the functional results gender was included as a co-variant to remove possible confounding by gender. Therefore the differences reported in the present study are in all likelihood, not related to gender.

### Psychometric data

The observation of a trend in higher anxiety-subscale (HADS) scores, in high-sensitization responders compared to low-sensitization responders, reaching significance in females, is intriguing, but should be interpreted with caution. First, the psychometric data belong to the secondary outcomes and as such are only of exploratory nature. Second, a number of comparisons of the psychometric variables were made, introducing a fairly high probability of a type I error. Third, the unequal gender distribution in the groups, may inadvertently have introduced a selection-bias. However, after carefully considering these caveats, the data in the present cohort may indicate that in females there is a correlation between scores from the anxiety subscale (HADS) and the magnitude of secondary hyperalgesia areas. Emotional and attentional mechanisms of pain processing, such as pain-related anxiety, catastrophizing and hyper-vigilance, are important elements of sensitivity to pain [53,54]. While it is reasonable to assume that high-sensitization responders are more sensitive to pin-prick noxious stimuli than low-sensitization responders, a recent well-powered experimental pain study indicates that hyper-vigilance, but not anxiety trait or catastrophizing behavior, predicts sensitivity to pain [53]. It should be noted that a very recent study demonstrated, that central sensitization can be modified volitionally by altering pain-related thoughts [55].

During central sensitization the central nervous system is regulated into a state of high reactivity, in which an uncoupling of the distinct simple stimulus-response relationship that defines nociceptive pain, occurs. Central sensitization thus changes, distorts or amplifies pain so it no longer matches the peripheral noxious stimuli, but instead is increased in severity, duration and spatial extent. Since the increased attention on central sensitization more than 30 years ago [56], much effort has been directed into understanding why some individuals are more prone to central sensitization. We wish to suggest that phenotypic expression of secondary hyperalgesia may act as a diagnostic tool to assess individual’s propensity to develop central sensitization. We have characterized variations in brain anatomy and central pain processing that are correlated with secondary hyperalgesia phenotype. We have furthermore established that these variations regarding spatial distribution of secondary hyperalgesia express differences in pain sensitivity leading to increased or augmented central responses regardless of unchanged pain scores and demonstrated a possible correlation to persistent pain states. The use of secondary hyperalgesia as a tool to phenotype individuals and thereby to establish their ability to develop central sensitization may be of predictive value in management of acute pain and in mitigating the trajectory to chronic pain.

## Conclusion

Individuals showing different phenotypic expressions of secondary hyperalgesia, a centrally mediated phenomenon, differ in brain morphology and in neuronal activation to noxious stimulation. Both the sensory-discriminative and the affective dimensions of pain are processed differently in subjects developing large and small areas of secondary hyperalgesia. This suggests differences in central sensitization across these phenotypes and may have predictive value on the susceptibility to development of acute and persistent pain.

## Supporting Information

S1 Table Individual demographic data. Table with individual demographic data for all included volunteers.
(PDF)

S2 Table Individual hemodynamic data. Table with individual hemodynamic data for all included volunteers.
(PDF)

S3 Table Individual pain ratings. Individual pain rating results after noxious mechanical stimulation at baseline (before burn injury), in the primary hyperalgesia area and in the secondary hyperalgesia area.
(PDF)

S4 Table Mechanical Pain Thresholds. Individual mechanical pain thresholds using using weighted-pin stimulators (PinPrick, MRC Systems, Heidelberg, Germany (8, 16, 32, 64, 128, 256, 512 mN)).
(PDF)

S5 Table HADS and PCS. Individual HADS and PCS results
(PDF)

## References

[1] Dirks J, Petersen KL, Rowbotham MC, Dahl JB (2002) Gabapentin suppresses cutaneous hyperalgesia following heat-capsaicin sensitization. Anesthesiology 97: 102–107. 10.1097/00000542-200207000-00015 12131110

[2] Petersen KL, Jones B, Segredo V, Dahl JB, Rowbotham MC (2001) Effect of remifentanil on pain and secondary hyperalgesia associated with the heat—capsaicin sensitization model in healthy volunteers. Anesthesiology 94: 15–20. 10.1097/00000542-200101000-00008 11135717

[3] Koppert W, Dern SK, Sittl R, Albrecht S, Schuttler J, et al. (2001) A new model of electrically evoked pain and hyperalgesia in human skin: the effects of intravenous alfentanil, S(+)-ketamine, and lidocaine. Anesthesiology 95: 395–402. 10.1097/00000542-200108000-00022 11506112

[4] Petersen KL, Rowbotham MC (1999) A new human experimental pain model: the heat/capsaicin sensitization model. Neuroreport 10: 1511–1516. 10.1097/00001756-199905140-00022 10380972

[5] Dahl JB, Brennum J, Arendt-Nielsen L, Jensen TS, Kehlet H (1993) The effect of pre- versus postinjury infiltration with lidocaine on thermal and mechanical hyperalgesia after heat injury to the skin. Pain 53: 43–51. 10.1016/0304-3959(93)90054-S 8316389

[6] Moiniche S, Dahl JB, Kehlet H (1993) Time course of primary and secondary hyperalgesia after heat injury to the skin. Br J Anaesth 71: 201–205. 10.1093/bja/71.2.201 8123392

[7] Werner MU, Petersen KL, Rowbotham MC, Dahl JB (2013) Healthy volunteers can be phenotyped using cutaneous sensitization pain models. PLoS One 8: e62733 10.1371/journal.pone.0062733 23671631 PMC3650051

[8] Ashburner J, Friston KJ (2000) Voxel-based morphometry—the methods. Neuroimage 11: 805–821. 10.1006/nimg.2000.0582 10860804

[9] Brooks J, Tracey I (2005) From nociception to pain perception: imaging the spinal and supraspinal pathways. J Anat 207: 19–33. 10.1111/j.1469-7580.2005.00428.x 16011543 PMC1571498

[10] Zambreanu L, Wise RG, Brooks JC, Iannetti GD, Tracey I (2005) A role for the brainstem in central sensitisation in humans. Evidence from functional magnetic resonance imaging. Pain 114: 397–407. 10.1016/j.pain.2005.01.005 15777865

[11] Ravn P, Frederiksen R, Skovsen AP, Christrup LL, Werner MU (2012) Prediction of pain sensitivity in healthy volunteers. J Pain Res 5: 313–326. 10.2147/JPR.S33925 23055774 PMC3442738

[12] Snaith RP, Zigmond AS (1986) The hospital anxiety and depression scale. Br Med J (Clin Res Ed) 292: 344 10.1136/bmj.292.6516.344 3080166 PMC1339318

[13] Sullivan MJ, Bishop SR, Pivik J (1995) The pain catastrophizing scale: development and validation. Psychological assessment 7: 524.

[14] Downie WW, Leatham PA, Rhind VM, Wright V, Branco JA, et al. (1978) Studies with pain rating scales. Ann Rheum Dis 37: 378–381. 10.1136/ard.37.4.378 686873 PMC1000250

[15] Torebjork HE, Lundberg LE, LaMotte RH (1992) Central changes in processing of mechanoreceptive input in capsaicin-induced secondary hyperalgesia in humans. J Physiol 448: 765–780. 1593489 10.1113/jphysiol.1992.sp019069 PMC1176227

[16] Dixon W (1991) Staircase bioassay: the up-and-down method. Neuroscience & Biobehavioral Reviews 15: 47–50. 10.1016/S0149-7634(05)80090-9 2052197

[17] Nichols T, Hayasaka S (2003) Controlling the familywise error rate in functional neuroimaging: a comparative review. Stat Methods Med Res 12: 419–446. 10.1191/0962280203sm341ra 14599004

[18] Eickhoff SB, Paus T, Caspers S, Grosbras MH, Evans AC, et al. (2007) Assignment of functional activations to probabilistic cytoarchitectonic areas revisited. Neuroimage 36: 511–521. 10.1016/j.neuroimage.2007.03.060 17499520

[19] Fischl B, Salat DH, Busa E, Albert M, Dieterich M, et al. (2002) Whole brain segmentation: automated labeling of neuroanatomical structures in the human brain. Neuron 33: 341–355. 10.1016/S0896-6273(02)00569-X 11832223

[20] Fischl B, Salat DH, van der Kouwe AJ, Makris N, Segonne F, et al. (2004) Sequence-independent segmentation of magnetic resonance images. Neuroimage 23 Suppl 1: S69–84. 10.1016/j.neuroimage.2004.07.016 15501102

[21] Fischl B, van der Kouwe A, Destrieux C, Halgren E, Segonne F, et al. (2004) Automatically parcellating the human cerebral cortex. Cereb Cortex 14: 11–22. 10.1093/cercor/bhg087 14654453

[22] Raz N, Lindenberger U, Rodrigue KM, Kennedy KM, Head D, et al. (2005) Regional brain changes in aging healthy adults: general trends, individual differences and modifiers. Cereb Cortex 15: 1676–1689. 10.1093/cercor/bhi044 15703252

[23] Latremoliere A, Woolf CJ (2009) Central sensitization: a generator of pain hypersensitivity by central neural plasticity. J Pain 10: 895–926. 10.1016/j.jpain.2009.06.012 19712899 PMC2750819

[24] Woolf CJ (2011) Central sensitization: implications for the diagnosis and treatment of pain. Pain 152: S2–15. 10.1016/j.pain.2010.09.030 20961685 PMC3268359

[25] Emerson NM, Zeidan F, Lobanov OV, Hadsel MS, Martucci KT, et al. (2013) Pain sensitivity is inversely related to regional grey matter density in the brain. Pain. 10.1016/j.pain.2013.12.004 PMC3944887 24333778

[26] Mountz JM, Bradley LA, Modell JG, Alexander RW, Triana-Alexander M, et al. (1995) Fibromyalgia in women. Abnormalities of regional cerebral blood flow in the thalamus and the caudate nucleus are associated with low pain threshold levels. Arthritis Rheum 38: 926–938. 10.1002/art.1780380708 7612042

[27] de Lange FP, Kalkman JS, Bleijenberg G, Hagoort P, van der Werf SP, et al. (2004) Neural correlates of the chronic fatigue syndrome—an fMRI study. Brain 127: 1948–1957. 10.1093/brain/awh225 15240435

[28] Robinson D, Wu H, Munne RA, Ashtari M, Alvir JM, et al. (1995) Reduced caudate nucleus volume in obsessive-compulsive disorder. Arch Gen Psychiatry 52: 393–398. 10.1001/archpsyc.1995.03950170067009 7726720

[29] Krishnan KR, McDonald WM, Escalona PR, Doraiswamy PM, Na C, et al. (1992) Magnetic resonance imaging of the caudate nuclei in depression. Preliminary observations. Arch Gen Psychiatry 49: 553–557. 10.1001/archpsyc.1992.01820070047007 1627046

[30] Chudler EH, Dong WK (1995) The role of the basal ganglia in nociception and pain. Pain 60: 3–38. 10.1016/0304-3959(94)00172-B 7715939

[31] Oshiro Y, Quevedo AS, McHaffie JG, Kraft RA, Coghill RC (2007) Brain mechanisms supporting spatial discrimination of pain. J Neurosci 27: 3388–3394. 10.1523/JNEUROSCI.5128-06.2007 17392455 PMC6672117

[32] Wunderlich AP, Klug R, Stuber G, Landwehrmeyer B, Weber F, et al. (2011) Caudate nucleus and insular activation during a pain suppression paradigm comparing thermal and electrical stimulation. Open Neuroimag J 5: 1–8. 10.2174/1874440001105010001 21643502 PMC3106353

[33] Gracely RH, Petzke F, Wolf JM, Clauw DJ (2002) Functional magnetic resonance imaging evidence of augmented pain processing in fibromyalgia. Arthritis Rheum 46: 1333–1343. 10.1002/art.10225 12115241

[34] Reyns N, Derambure P, Duhamel A, Bourriez JL, Blond S, et al. (2013) Motor cortex stimulation modulates defective central beta rhythms in patients with neuropathic pain. Clin Neurophysiol 124: 761–769. 10.1016/j.clinph.2012.10.011 23151426

[35] Aron AR, Robbins TW, Poldrack RA (2004) Inhibition and the right inferior frontal cortex. Trends Cogn Sci 8: 170–177. 10.1016/j.tics.2013.12.003 15050513

[36] Yan B, Li K, Xu J, Wang W, Li K, et al. (2005) Acupoint-specific fMRI patterns in human brain. Neurosci Lett 383: 236–240. 10.1016/j.neulet.2005.04.021 15876491

[37] Paulus MP, Feinstein JS, Leland D, Simmons AN (2005) Superior temporal gyrus and insula provide response and outcome-dependent information during assessment and action selection in a decision-making situation. Neuroimage 25: 607–615. 10.1016/j.neuroimage.2004.12.055 15784440

[38] Buckner RL, Andrews-Hanna JR, Schacter DL (2008) The brain’s default network: anatomy, function, and relevance to disease. Ann N Y Acad Sci 1124: 1–38. 10.1196/annals.1440.011 18400922

[39] Iannetti GD, Zambreanu L, Wise RG, Buchanan TJ, Huggins JP, et al. (2005) Pharmacological modulation of pain-related brain activity during normal and central sensitization states in humans. Proc Natl Acad Sci U S A 102: 18195–18200. 10.1073/pnas.0506624102 16330766 PMC1306794

[40] Baliki MN, Geha PY, Apkarian AV, Chialvo DR (2008) Beyond feeling: chronic pain hurts the brain, disrupting the default-mode network dynamics. J Neurosci 28: 1398–1403. 10.1523/JNEUROSCI.4123-07.2008 18256259 PMC6671589

[41] Bromm B (2001) Brain images of pain. News Physiol Sci 16: 244–249. 11572930 10.1152/physiologyonline.2001.16.5.244

[42] Peyron R, Schneider F, Faillenot I, Convers P, Barral FG, et al. (2004) An fMRI study of cortical representation of mechanical allodynia in patients with neuropathic pain. Neurology 63: 1838–1846. 10.1212/01.WNL.0000144177.61125.85 15557499

[43] Coghill RC, McHaffie JG, Yen YF (2003) Neural correlates of interindividual differences in the subjective experience of pain. Proc Natl Acad Sci U S A 100: 8538–8542. 10.1073/pnas.1430684100 12824463 PMC166264

[44] Pleger B, Tegenthoff M, Schwenkreis P, Janssen F, Ragert P, et al. (2004) Mean sustained pain levels are linked to hemispherical side-to-side differences of primary somatosensory cortex in the complex regional pain syndrome I. Exp Brain Res 155: 115–119. 10.1007/s00221-003-1738-4 15064892

[45] Veldhuijzen DS, Nemenov MI, Keaser M, Zhuo J, Gullapalli RP, et al. (2009) Differential brain activation associated with laser-evoked burning and pricking pain: An event-related fMRI study. Pain 141: 104–113. 10.1016/j.pain.2008.10.027 19058914 PMC6449044

[46] Wiech K, Seymour B, Kalisch R, Stephan KE, Koltzenburg M, et al. (2005) Modulation of pain processing in hyperalgesia by cognitive demand. Neuroimage 27: 59–69. 10.1016/j.neuroimage.2005.03.044 15978845

[47] Martinez V, Ben Ammar S, Judet T, Bouhassira D, Chauvin M, et al. (2012) Risk factors predictive of chronic postsurgical neuropathic pain: the value of the iliac crest bone harvest model. Pain 153: 1478–1483. 10.1016/j.pain.2012.04.004 22560289

[48] Salengros JC, Huybrechts I, Ducart A, Faraoni D, Marsala C, et al. (2010) Different anesthetic techniques associated with different incidences of chronic post-thoracotomy pain: low-dose remifentanil plus presurgical epidural analgesia is preferable to high-dose remifentanil with postsurgical epidural analgesia. J Cardiothorac Vasc Anesth 24: 608–616. 10.1053/j.jvca.2009.10.006 20005744

[49] Werner MU, Duun P, Kehlet H (2004) Prediction of postoperative pain by preoperative nociceptive responses to heat stimulation. Anesthesiology 100: 115–119; discussion 115A 10.1097/00000542-200401000-00020 14695732

[50] Skullerud K (1985) Variations in the size of the human brain. Influence of age, sex, body length, body mass index, alcoholism, Alzheimer changes, and cerebral atherosclerosis. Acta Neurol Scand Suppl 102: 1–94. 3887832

[51] Rijpkema M, Everaerd D, van der Pol C, Franke B, Tendolkar I, et al. (2012) Normal sexual dimorphism in the human basal ganglia. Hum Brain Mapp 33: 1246–1252. 10.1002/hbm.21283 21523857 PMC6870514

[52] Tang T, Jiao Y, Wang X, Lu Z (2013) Gender versus brain size effects on subcortical gray matter volumes in the human brain. Neurosci Lett 556: 79–83. 10.1016/j.neulet.2013.09.060 24103376

[53] Baum C, Huber C, Schneider R, Lautenbacher S (2011) Prediction of experimental pain sensitivity by attention to pain-related stimuli in healthy individuals. Percept Mot Skills 112: 926–946. 10.2466/02.09.22.PMS.112.3.926-946 21853779

[54] Lautenbacher S, Huber C, Schofer D, Kunz M, Parthum A, et al. (2010) Attentional and emotional mechanisms related to pain as predictors of chronic postoperative pain: a comparison with other psychological and physiological predictors. Pain 151: 722–731. 10.1016/j.pain.2010.08.041 20850220

[55] Salomons TV, Moayedi M, Erpelding N, Davis KD (2014) A brief cognitive-behavioural intervention for pain reduces secondary hyperalgesia. Pain. 10.1016/j.pain.2014.02.012 24569149

[56] Woolf CJ (1983) Evidence for a central component of post-injury pain hypersensitivity. Nature 306: 686–688. 10.1038/306686a0 6656869

