# Correction: Secondary Hyperalgesia Phenotypes Exhibit Differences in Brain Activation during Noxious Stimulation

**DOI:** 10.1371/journal.pone.0128640

**Published:** 2015-05-15

**Authors:** Mohammad Sohail Asghar, Manuel Pedro Pereira, Mads Utke Werner, Johan Mårtensson, Henrik B. W. Larsson, Jørgen Berg Dahl

In the Author Contributions section, Manuel Pedro Pereira (MPP) should be listed as one of the persons who wrote the paper.
