# Supplementary material for: Secondary Hyperalgesia Phenotypes Exhibit Differences in Brain Activation during Noxious Stimulation
Source: PLoS One. 2015 Jan 23;10(1):e0114840. doi: 10.1371/journal.pone.0114840 (PMC4304709; doi:10.1371/journal.pone.0114840)
Supplement: S1 Table — Table with individual demographic data for all included volunteers. (PDF) [file pone.0114840.s001.pdf]

Subject no	Sex	Group	Dominant hand	Age	Height	Weight
H1	2	1	R	25,210959	180	75
L1	2	2	R	24,405479	172	65
L2	1	2	R	22,873973	184	83
H2	1	1	R	23,035616	186	80
H3	2	1	R	25,364384	164	60
H4	1	1	R	22,649315	181	74
H5	2	1	R	25,358904	175	68
H6	2	1	R	23,589041	165	63
H7	1	1	L	23,742466	188	80
L3	1	2	R	22,879452	188	70
H8	1	1	R	23,610959	180	70
H9	2	1	R	21,693151	170	63
H10	2	1	R	21,80274	165	58
H11	2	1	R	24,120548	174	73
L4	2	2	R	31,175342	163	55
H12	1	1	R	31,506849	182	75
L5	1	2	R	27,578082	177	71
H13	2	1	R	23,531507	165	65
H14	2	1	R	23,232877	157	50
H15	2	1	R	23,175342	169	69
L6	1	2	R	21,052055	195	81
L7	1	2	R	22,369863	184	79
H16	1	1	R	25,40274	190	83
L8	1	2	R	29,378082	196	95
H17	2	1	R	28,70137	183	73
H18	2	1	R	22,816438	185	73
L9	1	2	R	22,060274	182	80
L10	2	2	R	23,29863	165	61
H19	2	1	R	22,846575	172	62
L11	1	2	L	21,506849	173	64
L12	1	2	R	26,550685	165	64
L13	1	2	R	22,810959	188	82
L14	2	2	R	21,794521	160	65
H20	2	1	R	24,816438	174	60
L15	2	2	R	25,194521	168	62
L16	1	2	R	23,723288	186	77
L17	2	2	R	24,493151	176	70
L18	1	2	R	25,106849	196	78
L19	2	2	R	23,243836	165	56
L20	2	2	R	23,4	175	72

H: High-sensitization responders
L: Low-sensitization responders

sex
1: Male
2: Female

R: Right handed
L: Left handed
