# Supplementary material for: Secondary Hyperalgesia Phenotypes Exhibit Differences in Brain Activation during Noxious Stimulation
Source: PLoS One. 2015 Jan 23;10(1):e0114840. doi: 10.1371/journal.pone.0114840 (PMC4304709; doi:10.1371/journal.pone.0114840)
Supplement: S2 Table — Table with individual hemodynamic data for all included volunteers. (PDF) [file pone.0114840.s002.pdf]

Subject No	Sex	Group	T1sys	T2sys	T3sys	T4sys	T5sys	T6sys
H1	2	1	116	117	115	126	124	122
L1	2	2						
L2	1	2		134	133	145	135	136
H2	1	1	153	141	142			146
H3	2	1	120	116	114	117	117	133
H4	1	1	134	131	134	130	133	124
H5	2	1	122	116	121			
H6	2	1	115	118	121	120	127	121
H7	1	1	125	124	135	135	133	142
L3	1	2	133	108	107		115	115
H8	1	1	112	113	114			110
H9	2	1	116	114	112	116		122
H10	2	1	121	118	119	121		123
H11	2	1	114	108	114	116	120	112
L4	2	2	126	125	128	126		129
H12	1	1	116	108	113	111		110
L5	1	2	135	123	126	126	127	128
H13	2	1	118	115	116	116	114	129
H14	2	1	120	121	123	122	139	120
H15	2	1	126	125	122	127	125	120
L6	1	2	128	110	116	113		121
L7	1	2	123	121	127	124	125	129
H16	1	1	132	125	124	130		142
L8	1	2	111	109	108	109	110	101
H17	2	1	125	122	123	120	122	120
H18	2	1	140	139	138	137	140	139
L9	1	2	126	124	125	126		131
L10	2	2	118	121	118	117	117	116
H19	2	1	124	118	115	121	118	116
L11	1	2	128	118	123	123	124	133
L12	1	2	129	127	121	127	122	110
L13	1	2	134		132	132		
L14	2	2	102	101	104	100	103	119
H20	2	1	124	126	127	126	132	125
L15	2	2	112	114	110	120	121	112
L16	1	2	125	122	125	125	122	133
L17	2	2	127	116	116	117	116	113
L18	1	2	114	116	118	118	120	121
L19	2	2	130	130	122	122	122	132
L20	2	2	140	120	121	123	123	122

H: High sensitization responders
L: Low sensitization responders

sex:
1: Male
2: Female

Group
1: High sensitization responders
2: Low sensitization responders

Sys: Systolic blood pressure
Dia: Diastolic blood pressure
Pulse: Heart rate

Co2: End-Tidal Co2

T7sys	T8sys	T9sys	T10sys	T1dia	T2dia	T3dia	T4dia	T5dia
124	123	124	125	72	72	70	75	76
135	133	138	135		65	62	72	72
149	158	144	148	83	72	68		
129	123	125	124	69	66	64	67	78
127	128	118	122	71	66	70	62	67
123	122	123	123	58	54	66		
118	120	120	120	75	69	70	67	71
145	105	130	137	79	72	82	83	84
113	115	116	120	84	64	62		68
117	110	117	119	61	57	58		
121	118	124	124	57	59	58	68	
116	115	112	122	77	73	74	72	
111	111	114	108	63	63	63	65	82
124	113	114	117	75	79	74	75	
116	113	113	110	71	71	72	72	
123	126	123	125	74	66	61	66	66
125	117	127	122	76	67	70	65	70
112	119	116	115	69	79	85	88	95
117	116	120	121	75	79	76	78	74
118	117	119	124	66	61	53	54	
153	123	126	128	62	67	71	61	62
136	133	136	142	68	68	62	69	
103	100	100	101	61	60	62	67	69
116	117	114	121	61	59	58	57	59
133	134	133	144	77	75	66	69	72
135	128	140	136	57	56	59	60	
114	112	115	113	63	63	61	61	67
112	111	117	125	70	71	72	70	70
123	128	121	127	73	64	69	68	68
120	120	119	118	70	62	69	66	64
	118	118		67		67	62	
120	118	115	123	57	55	58	55	60
126	125	126	126	80	68	64	66	69
111	108	106	108	66	67	66	61	71
132	130	132	127	63	60	59	61	60
113	112	112	113	74	74	68	66	66
119	114	126	122	56	58	55	56	56
122	123	125	125	73	100	70	69	72
144	136	137	134	79	73	76	73	72

T6dia	T7dia	T8dia	T9dia	T10dia	T1pulse	T2pulse	T3pulse	T4pulse
68	73	70	77	78	72	70	70	75
68	71	67	68	68		45	39	44
71	62	62	70	74	61	55	47	
67	60	65	74	72	65	61	64	68
72	60	60	64	72	49	61	54	56
	70	66	76	68	65	68	67	
67	67	66	70	75	62	60	57	66
70	64	60	67	64	57	57	56	57
61	63	65	70	68	72	70	71	61
70	62	67	70	69	72	71	71	
61	57	60	61	64	54	51	55	58
76	77	75	76	87	85	72	73	67
65	69	66	64	69	48	50	55	53
77	72	67	71	72	62	63	60	57
42	71	66	64	65	75	71	72	73
65	62	63	64	85	45	48	46	49
72	63	59	56	64	71	69	73	71
79	64	64	62	65	61	71	75	63
74	72	75	77	77	67	75	78	79
57	55	58	59	62	72	59	59	59
63	42	51	61	64	58	55	50	48
69	72	68	69	65	59	67	66	72
56	60	56	61	60	41	42	43	51
81	60	63	64	62	54	54	54	59
69	74	70	71	63	58	53	51	51
66	68	68	66	64	60	60	63	57
59	63	61	61	58	59	56	54	51
68	71	67	65	72	59	58	65	61
79	71	68	74	72	85	74	78	71
67	61	62	67	71	88	91	84	95
		62	62		58	59	58	59
62	62	63	60	60	71	58	57	58
68	66	64	66	68	64	60	61	59
67	64	66	65	70	71	66	63	57
64	57	63	57	57	37	38	38	38
66	61	63	65	65	64	61	60	59
65	56	58	56	60	65	65	63	60
65	66	64	67	69	47	57	52	57
71	68	67	72	80	58	52	58	66

T5pulse	T6pulse	T7 pulse	T8 pulse	T9pulse	T10pulse	T1co2	T2co2	T3co2
76	68	73	70	77	78	4,4	4,2	4,6
43	45	44	51	50	53		4,7	4,9
	52	43	44	38	53	4,2	4,6	4,2
71	69	71	62	76	74	4,8	4,6	4,7
58	53	65	55	54	54	4,8	4,5	4,9
		66	64	66	71	4,7	4,7	4,6
53	51	59	51	65	63	4,5	4,7	4,7
61	66	56	55	66	58	5	5,1	5,1
75	65	63	61	80	72	4,8	4,7	4,7
	72	74	72	74	73	5,6	5,4	5,5
	51	50	50	54	50	4,2	3,9	3,9
	74	65	76	72	69	4,3	4,6	4,3
51	67	58	54	48	57	4,3	4,2	4,3
	61	65	68	69	70	5,4	5,3	5,5
	79	68	75	72	70	5,4	5,1	5,3
53	48	48	51	55	67	5,3	5,6	5,6
72	73	72	72	71	71	4,5	4,5	4,5
91	64	70	54	57	76	5,1	5,2	5
82	73	71	74	75	81	4,2	4,1	4,2
	62	56	57	55	57	4,5	5	5
51	47	49	50	51	68	5	4,8	5,1
	48	61	62	58	63	5,1	5,1	5,3
50	53	57	60	57	55	5,6	5,5	5,5
58	62	61	59	60	61	5,1	5,4	5,4
52	54	56	57	55	61	4,7	4,8	4,9
	67	67	67	70	67	5,3	5,2	5,2
53	65	58	56	58	60	4,6	4,6	4,5
56	62	64	63	58	60	4,6	4,7	4,9
74	75	76	68	82	83	4,8	5	4,9
91	85	80	85	76	90	5,3	5,6	5,6
	56	57	55	61	56	5	5	4,9
66	59	59	61	68	63	4,9	5	5
72	64	66	65	64	65	4,5	4,7	4,5
69	67	69	67	67	62	4,9	4,6	4,7
41	42	36	41	39	41	5,5	5,4	5,5
58	58	59	56	56	61	5	5	5,2
59	61	64	58	62	59	5,1	5	5,4
53	58	64	70	71	70			3,5
64	71	69	70	69	68	4,6	4,5	4,8

T4co2	T5co2	T6co2	T7co2	T8co2	T9co2	T10co2
4,6	4,7	4,3	4,4	4,5	4,6	4,4
4,7	5,1	5,1	5	5	4,8	5
		4	4	4,3	4,2	4,5
4,8	4,7	4,2	4,4	4,4	4,6	4,4
5,1	4,6	4,6	4,8	5	4,8	4,6
			4,8	4,9	4,9	4,8
4,3	4,5	4				
5,3	5,2	4,7	4,9	4,9	4,8	4,9
4,6		4,6		4,2	4,2	4,4
		5,3	5,5	5,6	5,4	5,5
3,8		3,6	3,9	3,7	3,7	3,7
4,5		4,5	4,8	4,7	4,8	4,8
4,5	4,2	3,9	4	4,2	4	4,3
5,3		5,3	5,2	5,2	5,1	5,1
5,4		5,3	5,4	5,5	5,4	5,2
5,5	5,5	5,3	5,4	5,4	5,6	5,4
4,6	4,6	4,5	4,2	4,6	4,6	4,6
5,1	5	5,4	5,3	5,4	5,4	5,4
4,4	4,1	3,6	3,5	3,4		3,7
4,7		5,1	5,1	5,2	5,2	5,1
5,1	4,7	4,7	5	5	5,1	4,8
5		5,1	4,9	5	4,9	5,1
5,4	5,5	5,3	5,4	5,2	5,3	5,2
5,4	5,4	5,4	5,3	5,4	5,5	5,4
4,7	4,8	4,9	4,8	4,9	4,6	4,7
5,5		5,5	5,3	5,4	5,4	5,2
4,5	4,5	4,6	4,3	4,6	4,5	4,5
4,9	4,8					
5	5,2	4,5	4,7	4,9	4,8	4,7
5,5	5,6	5,5	5,3	5,5	5,5	5,4
5,1		4,9	5,1	5	5	5,1
5,1	4,9	4,9	4,9	5	4,9	4,7
4,9	4,7	3,9	3,8	4	4,2	4,1
4,9	4,5	4,4	4,6	4,7	4,9	4,5
5,5	5,3	5,3	5,3	5,4	5,5	5,4
5,2	5	5	5,2	4,9	5	5
5,3	5,4	5,1	5,2	5,2	5,3	5
3,8			4,8	4,8	5,1	4,8
4,5	4,6	4,5	4,6	4,6	4,8	4,7
