# Supplementary material for: Secondary Hyperalgesia Phenotypes Exhibit Differences in Brain Activation during Noxious Stimulation
Source: PLoS One. 2015 Jan 23;10(1):e0114840. doi: 10.1371/journal.pone.0114840 (PMC4304709; doi:10.1371/journal.pone.0114840)
Supplement: S3 Table — Individual pain rating results after noxious mechanical stimulation at baseline (before burn injury), in the primary hyperalgesia area and in the secondary hyperalgesia area. (PDF) [file pone.0114840.s003.pdf]

Data #	NRS_BL	NRS_SH	NRS_PH
H1	2	3	4
L1	2,5	7,5	7
L2	4	3	5
H2	2	3	5
H3	3	5	6
H4	3	4	7
H5	2	4	6
H6	5	4	7
H7	3	7	8
L3	4	7	8
H8	1	4	4
H9	2	3,5	6
H10	7	10	10
H11	3	4	4
L4	3	5	5
H12	6	6	6
L5	3	5	6
H13	1	5	6
H14	5	5	9
H15	4	7	9
L6	4	7	7,5
L7	2	4	4
H16	3	2	3
L8	1	1	3
H17	5	6	7
H18	5	6	7
L9	3	6,5	7
L10	7	4,5	7,5
H19	2	2	3
L11	2	3	2
L12	4	4	5
L13	4	4,5	4,5
L14	4	7	8
H20	7	8,5	9,5
L15	1	3	3
L16	2,5	2	5
L17	1,5	3	3,5
L18	6	7	8
L19	0	1	1
L20	0	2	2

H: High-sensitization Responders
L: Low-sensitization Responders

NRS_BL: Numeric Rating Scale after noxious stimulation at baseline
NRS_SH: Numeric Rating Scale after noxious stimulation at Secondary hyperalgesia area
NRS_PH: Numeric Rating Scale after noxious stimulation at Primary hyperalgesia area
