# Supplementary material for: Secondary Hyperalgesia Phenotypes Exhibit Differences in Brain Activation during Noxious Stimulation
Source: PLoS One. 2015 Jan 23;10(1):e0114840. doi: 10.1371/journal.pone.0114840 (PMC4304709; doi:10.1371/journal.pone.0114840)
Supplement: S4 Table — Individual mechanical pain thresholds using using weighted-pin stimulators (PinPrick, MRC Systems, Heidelberg, Germany (8, 16, 32, 64, 128, 256, 512 mN)). (PDF) [file pone.0114840.s004.pdf]

Data #	Sex	Group	PPT_M	PPT_converted	Data #
H1	2	1	32	3	L1
H2	1	1	128	5	L2
H3	2	1	256	6	L3
H4	1	1	256	6	L4
H5	2	1	512	7	L5
H6	2	1	128	5	L6
H7	1	1	128	5	L7
H8	1	1	128	5	L8
H9	2	1	512	7	L9
H10	2	1	128	5	L10
H11	2	1	256	6	L11
H12	1	1	128	5	L12
H13	2	1	128	5	L13
H14	2	1	256	6	L14
H15	2	1	128	5	L15
H16	1	1	256	6	L16
H17	2	1	64	4	L17
H18	2	1	64	4	L18
H19	2	1	256	6	L19
H20	2	1	512	7	L20

H: High-sensitization Responders
L: Low-sensitization Responders

PPT_M: Weighted pins absol
PPT_concerted: Weighted pi

Sex
1: Male
2: Female

Group
1: High-sensitization responders
2: Low-sensitization responders

Sex	Group	PPT_M	PPT_converted
2	2	512	7
1	2	128	5
1	2	512	7
2	2	256	6
1	2	128	5
1	2	128	5
1	2	128	5
1	2	128	5
1	2	512	7
2	2	256	6
1	2	64	4
1	2	128	5
1	2	128	5
2	2	512	7
2	2	256	6
1	2	256	6
2	2	512	7
1	2	128	5
2	2	512	7
2	2	256	6

lute values
ins converted values
