# Supplementary material for: Secondary Hyperalgesia Phenotypes Exhibit Differences in Brain Activation during Noxious Stimulation
Source: PLoS One. 2015 Jan 23;10(1):e0114840. doi: 10.1371/journal.pone.0114840 (PMC4304709; doi:10.1371/journal.pone.0114840)
Supplement: S5 Table — Individual HADS and PCS results (PDF) [file pone.0114840.s005.pdf]

		HADS		PCS			
**H/L**	**Volunteer #**	**Anxiety**	**Depression**	**Rumination**	**Maginificatic**	**Helplessness**	**PCS_Total**
H	1	1	1	1	0	0	1
H	2	0	0	3	2	2	7
H	3	5	1	6	1	4	11
H	4	4	3	4	2	5	11
H	5	2	3	7	2	3	12
H	6	5	0	2	1	1	4
H	7	4	1	6	3	2	11
H	8	5	7	9	4	4	17
H	9	6	4	0	0	2	2
H	10	7	3	14	7	15	36
H	11						
H	12	0	0	4	2	3	9
H	13	15	4	10	7	7	24
H	14	5	3	4	1	5	10
H	15	8	6	8	9	8	25
H	16	1	2	5	1	1	7
H	17	9	2	9	6	9	24
H	18	8	2	5	2	3	10
H	19	8	1	5	3	6	14
H	20	2	1	5	0	4	9
L	21	0	1	4	0	0	4
L	22	2	1	4	1	1	6
L	23	4	1	7	3	3	13
L	24	5	1	3	5	7	15
L	25	0	0	0	0	0	0
L	26	5	3	3	6	3	12
L	27	4	5	4	2	2	8
L	28	0	3	5	3	5	13
L	29	8	3	4	0	1	5
L	30	1	3	4	1	8	13
L	31	2	1	3	2	2	7
L	32	4	2	4	2	1	7
L	33	4	0	4	2	4	10
L	34	1	1	6	3	9	18
L	35						
L	36	5	1	1	0	1	2
L	37	4	1	2	2	2	6
L	38	5	9	7	3	4	14
L	39	2	0	1	1	2	4
L	40	4	2	2	1	1	4

H: High-sensitization responders

L: Low-sensitization responders
